# ProNodal acts via FGFR3 to govern duration of Shh expression in the prechordal mesoderm

**DOI:** 10.1242/dev.119628

**Published:** 2015-11-15

**Authors:** Pamela S. Ellis, Sarah Burbridge, Sandrine Soubes, Kyoji Ohyama, Nadav Ben-Haim, Canhe Chen, Kim Dale, Michael M. Shen, Daniel Constam, Marysia Placzek

**Affiliations:** 1 The Bateson Centre andDepartment of Biomedical Science, University of Sheffield, Western Bank, Sheffield S10 2TN, UK; 2 ISREC, School of Life Sciences, Ecole Polytechnique Fédérale de Lausanne, Epalinges CH 1066, Switzerland; 3 Departments of Medicine and Genetics & Development, Columbia University Medical Center, Herbert Irving Comprehensive Cancer Center, 1130 St. Nicholas Avenue, New York, NY 10032, USA; 4 Institute of Molecular and Cell Biology, 61 Biopolis Drive, Proteos, Singapore 138673, Singapore; 5 College of Life Sciences, University of Dundee, Dundee DD1 5EH, UK

**Keywords:** BMP, Nodal, Sonic hedgehog, Forebrain ventral midline, Prechordal mesoderm

## Abstract

The secreted glycoprotein sonic hedgehog (Shh) is expressed in the prechordal mesoderm, where it plays a crucial role in induction and patterning of the ventral forebrain. Currently little is known about how Shh is regulated in prechordal tissue. Here we show that in the embryonic chick, Shh is expressed transiently in prechordal mesoderm, and is governed by unprocessed Nodal. Exposure of prechordal mesoderm microcultures to Nodal-conditioned medium, the Nodal inhibitor CerS, or to an ALK4/5/7 inhibitor reveals that Nodal is required to maintain both Shh and *Gsc* expression, but whereas *Gsc* is largely maintained through canonical signalling, Nodal signals through a non-canonical route to maintain Shh. Further, Shh expression can be maintained by a recombinant Nodal cleavage mutant, proNodal, but not by purified mature Nodal. A number of lines of evidence suggest that proNodal acts via FGFR3. ProNodal and FGFR3 co-immunoprecipitate and proNodal increases FGFR3 tyrosine phosphorylation. In microcultures, soluble FGFR3 abolishes Shh without affecting *Gsc* expression. Further, prechordal mesoderm cells in which *Fgfr3* expression is reduced by *Fgfr3* siRNA fail to bind to proNodal. Finally, targeted electroporation of *Fgfr3* siRNA to prechordal mesoderm *in vivo* results in premature Shh downregulation without affecting *Gsc*. We report an inverse correlation between proNodal-FGFR3 signalling and pSmad1/5/8, and show that proNodal-FGFR3 signalling antagonises BMP-mediated pSmad1/5/8 signalling, which is poised to downregulate *Shh*. Our studies suggest that proNodal/FGFR3 signalling governs Shh duration by repressing canonical BMP signalling, and that local BMPs rapidly silence Shh once endogenous Nodal-FGFR3 signalling is downregulated.

## Abstract (precis)

**Highlighted article:** In the chick prechordal mesoderm, the Nodal precursor proNodal acts via a non-canonical route to inhibit BMP signalling and thus maintain Shh expression

## INTRODUCTION

In the developing vertebrate embryo, ventral midline cells of the neural tube are underlain by a cellular rod of axial mesoderm, composed of prechordal mesoderm beneath the prospective forebrain, and notochord beneath the prospective spinal cord and hindbrain (Adelmanm, 1922; Spemann and Mangold, 1924). The spatial relationship between prechordal mesoderm and ventral midline cells of the developing forebrain (rostral diencephalic ventral midline, RDVM), studied in detail in the chick embryo, is highly dynamic (Dale et al., 1999; Seifert et al., 1993). Differential tissue movements, and the rapid proliferation of forebrain ventral neural tissue, results in the prechordal mesoderm being in register, in turn, with prospective anterior/tuberal hypothalamus, posterior (mammillary) hypothalamus, then caudal diencephalon (Dale et al., 1999; Pearson and Placzek, 2013). However, studies in a range of vertebrates suggest that even such transient apposition is sufficient for the prechordal mesoderm to induce the RDVM (Aoto et al., 2009; Dale et al., 1997; Geng et al., 2008; Li et al., 1997; Mathieu et al., 2002; Patten et al., 2003; Pearson and Placzek, 2013; Pera and Kessel, 1997; Schneider and Mercola, 1999) and to contribute to the patterning of diverse forebrain ventral cell types, including those in the ventral telencephalon, pre-optic area, hypothalamus and thalamus (Aoto et al., 2009; Epstein, 2012; García-Calero et al., 2008; Geng et al., 2008; Kiecker and Niehrs, 2001; Li et al., 1997; Ohyama et al., 2005; Pera and Kessel, 1997; Puelles et al., 2012; Shimamura and Rubenstein, 1997; Szabo et al., 2009).

Prechordal mesoderm, like notochord, expresses the secreted glycoprotein, sonic hedgehog (Shh) (Patten and Placzek, 2000; Shimamura et al., 1995). Shh acts as an autocrine factor, preventing apoptosis of the prechordal mesoderm and promoting its survival (Aoto et al., 2009). Additionally, Shh deriving from the prechordal mesoderm appears to be instrumental in mediating, or contributing to, its inducing and patterning activities. Embryos in which Shh expression is lost show failure of RDVM induction and optic field separation, and consequent holoprosencephalic phenotypes (Belloni et al., 1996; Chiang et al., 1996; Patten and Placzek, 2000; Roessler et al., 1996; Roessler and Muenke, 2010). In mouse embryos, Shh from the prechordal plate synergises with Six3 to activate Shh expression in the RDVM (Geng et al., 2008; Shimamura and Rubenstein, 1997). Mouse embryos in which Shh is conditionally deleted in the RDVM, but not in the prechordal mesoderm, do not have cyclopia, and express the ventral forebrain marker, Nkx2.1 (Szabo et al., 2009). Studies of isolated tissue explants, in which the role of prechordal mesoderm-derived Shh can be specifically tested, reveal that Shh from the prechordal mesoderm is required to induce RDVM-like cells (Dale et al., 1997; Patten et al., 2003) and pattern ventral forebrain-like cells (Ohyama et al., 2005).

The precise regulation and duration of Shh expression in prechordal mesoderm appears to be crucial to a number of its functions. Loss of a single copy of *Shh*, or mutations that lead to reduced expression of Shh in prechordal mesoderm, result in holoprosencephaly (Belloni et al., 1996; Geng et al., 2008; Leung et al., 2010; Roessler et al., 1996; Roessler and Muenke, 2010). The temporal perturbation of Shh signalling correlates with the severity of holoprosencephalic phenotypes: the earlier the alteration, the more severe the phenotype (Cordero et al., 2004; Mercier et al., 2013; Zhang et al., 2006). Premature inactivation of Shh within the prechordal mesoderm prevents the development of Nkx2.1^+^/Shh^+^ hypothalamic-like progenitor cells (Ohyama et al., 2005). Together, these studies emphasise the functional significance, and importance of the tight temporal control of Shh in the prechordal mesoderm. Despite this progress, little is known about how Shh is regulated there. Cis-acting sequences that regulate *Shh* in the mouse prechordal plate have been identified (Jeong et al., 2006) but to date, in-depth analyses have focused on enhancer elements that direct expression in the notochord and ventral neural tube, rather than the prechordal mesoderm (Epstein et al., 1999; Geng et al., 2008; Jeong et al., 2006; Muller et al., 1999; Trowe et al., 2013).

The TGFβ superfamily member, Nodal, is expressed within the chick prechordal mesoderm. Nodal is known to govern early axial mesoderm, including prechordal mesoderm development (Chen and Schier, 2001; Dougan et al., 2003; Green et al., 1992; Gurdon et al., 1996; Hatta et al., 1991; Lowe et al., 2001; Thisse et al., 1994). Further, previous studies have suggested an interaction between Nodal and Shh signalling pathways in RDVM induction and forebrain patterning (Mathieu et al., 2002; Mercier et al., 2013; Patten et al., 2003; Placzek and Briscoe, 2005; Rohr et al., 2001; Taniguchi et al., 2012). Together, this prompted us to address whether Nodal temporally regulates Shh in the prechordal mesoderm. Here we show that Shh and *Nodal* are co-expressed in the prechordal mesoderm, that Nodal maintains Shh expression and that Shh is silenced concomitant with the loss of *Nodal* expression. Unexpectedly, our results reveal that maintenance of Shh by Nodal is mediated by its precursor, proNodal. Our studies show that proNodal does not operate through the canonical Nodal-ALK pathway, but instead binds and activates FGFR3. Targeted knockdown of *Fgfr3* results in a failure of cells to bind to proNodal and *in vivo*, results in premature downregulation of Shh, without affecting expression of *Gsc*, a defining marker of prechordal mesoderm. We demonstrate that the duration of Shh expression is dependent on the antagonistic actions of proNodal-FGFR3 and BMP. *Shh/*Shh is downregulated by BMP signalling and endogenous proNodal-FGFR3 inhibits the BMP pathway to maintain Shh expression. Together with previous studies our findings suggest that Nodal signals through a canonical pathway to induce prechordal mesoderm, and through an FGF signalling pathway to regulate Shh expression. This latter mechanism is deployed to tightly regulate Shh at a time that is crucial to forebrain development.

## RESULTS

### Nodal signals via canonical and non-canonical routes in prechordal mesoderm

**Fig. 1.**
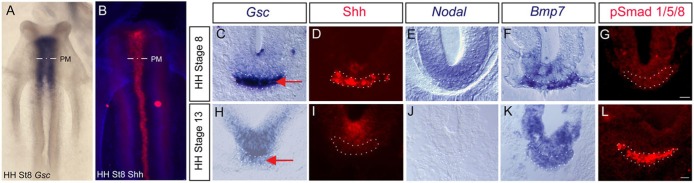
**Dynamic changes in prechordal mesoderm.** (A,B) Wholemount views of st8 chick embryos after *in situ* hybridisation to detect *Gsc* or immunohistochemistry to detect Shh. (C-L) Transverse sections at the level of prechordal mesoderm (PM in A,B) showing expression of Shh and pSmad1/5/8 protein by immunohistochemistry and *Gsc*, *Nodal* and *Bmp7* by *in situ* hybridisation at st8 or st13 (dots outline prechordal mesoderm; red arrows point to prechordal mesoderm in (C,H). Scale bar: 25 µm.

The transcription factor *Gsc* is a well-characterised marker of chick prechordal mesoderm, where it is expressed from gastrula stages (Izpisúa-Belmonte et al., 1993). Strong expression persists in prechordal mesoderm until Hamburger–Hamilton stage (HH st)8 (Fig. 1A,C). After this, *Gsc* expression begins to decline, and by HH st13, can be detected only weakly (Fig. 1H, red arrow). Shh and *Nodal* are both detected in the HH st8 prechordal mesoderm, then decline, and are not detected at all by HH st13 (Fig. 1B,D,E,I,J). Previous studies in a wide range of other vertebrates have shown that Nodal signalling is necessary and sufficient for *Gsc* expression in the prechordal mesoderm (Erter et al., 1998; Feldman et al., 1998; Ro and Dawid, 2010; Vincent et al., 2003). This, and the correlation in expression patterns that we detect in the chick, led us to ask whether Nodal signalling governs the temporal duration of both *Gsc* and Shh expression within the chick prechordal mesoderm.

**Fig. 2.**
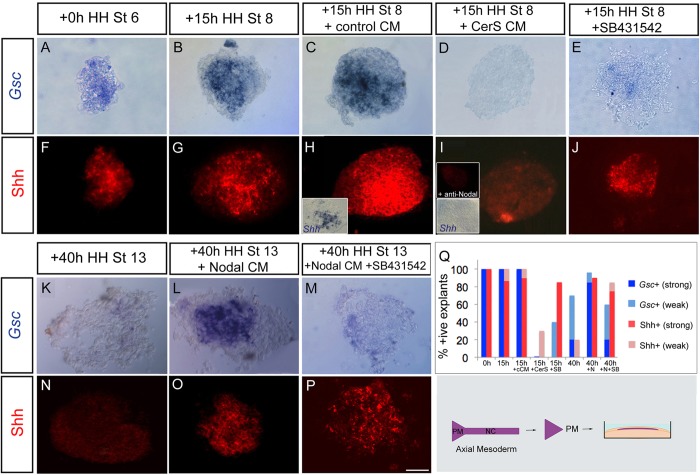
**Canonical and non-canonical Nodal signalling governs prechordal mesoderm characteristics.** (A-P) Analysis of *Gsc* and Shh in st6 prechordal mesoderm microcultures (shown in schematic), acutely (A,F), after culture for 15 h (st8 equivalent) (B-E,G-J) or after culture for 40 h (st13 equivalent) (K-P), exposed to control conditioned medium (CM) (A-C,F-H,K,N), to CerS CM (D,I), to Nodal CM (L,O), the ALK4/5/7 inhibitor, SB431542 (E,J) or Nodal and SB431542 (M,P). Representative examples are shown for robust (A,B,C,L), weak (E,K,M) or no (D) *Gsc* expression. Representative examples are shown for robust (F-H,J,O,P), residual patchy (I) or no (N) Shh expression. Insets in I show prechordal mesoderm incubated in anti-Nodal antibody (top inset) or CerS CM (bottom inset). (Q) Quantitative analyses, showing summed data from *n*=8-12 explants/condition. cCM, control conditioned medium; CerS, Cerberus-short; SB, SB431542; N, Nodal. Scale bar: 50 µm.

The small size of the prechordal mesoderm means that *in vivo* manipulation is difficult, and we therefore developed an *ex vivo* microculture assay, in which prechordal mesoderm (together with underlying endoderm: hereafter termed prechordal mesendoderm) (Fig. 2, schematic) is isolated at HH st6/7 and cultured until the equivalent of HH st8 (15 h time point) or st13 (40 h time point). In this situation, both acutely dissected prechordal mesendoderm explants, and those cultured until HH st8, express *Gsc* and Shh (Fig. 2A,B,F,G,Q), whereas explants cultured to the later, st13, time point show weak or no expression of *Gsc* and fail to robustly maintain Shh (Fig. 2K,N,Q). These observations indicate that expression of *Gsc* and Shh *ex vivo* follow the same kinetics as is observed in intact embryos. Using this assay, we asked whether Nodal is required for maintenance of both *Gsc* and Shh, by culturing explants in the presence of the Nodal inhibitor, Cerberus-short (Cer-S), a secreted cysteine-knot protein. Short-term cultured prechordal mesoderm explants incubated with Cer-S-transfected cell supernatant, but not mock-transfected cell supernatant, show a marked downregulation of *Gsc*, and both Shh mRNA and protein (Fig. 2C,D,H,I,Q). An anti-Nodal antibody is similarly effective in downregulating Shh (Fig. 2I, top inset). At the same time, we used the assay to ask whether provision of exogenous Nodal can prolong Shh expression in prechordal mesendoderm. Western blot analysis of Nodal-conditioned medium (Nodal-CM), from HEK293T cells transiently transfected with mouse Nodal, revealed a mixture of uncleaved and mature Nodal (Fig. S1). When HH st6/7 prechordal mesendoderm explants are exposed to mock-transfected medium and cultured to the equivalent of HH st13, explants behave in the same way as controls, i.e. weakly expressing *Gsc* but not Shh (data not shown). By contrast, tissue exposed to Nodal-CM robustly expresses both *Gsc* and Shh (Fig. 2L,O,Q). Thus, Nodal can prolong Shh expression in prechordal mesoderm.

A wealth of data shows that in other species, Nodal signals through the activin receptor to govern *Gsc* expression (Whitman, 2001). We therefore asked whether Nodal signals through the activin receptor to maintain *Gsc* and Shh in chick prechordal mesoderm. HH st6 prechordal mesendoderm explants were cultured to a st8 equivalent, in the presence of the ALK4/5/7 receptor inhibitor, SB431542, or to a st13 equivalent in the presence of Nodal-CM and SB431542. As anticipated, *Gsc* expression fails to be maintained after inhibition of canonical ALK-mediated Nodal signalling (Fig. 2E,M,Q). Surprisingly, however, robust expression of Shh persists in the prechordal mesoderm in the presence of SB431542 (Fig. 2J,P,Q). Together, this data suggests that in prechordal mesoderm, Nodal signals via a canonical route to govern *Gsc* expression, but is required to signal through a non-canonical route to govern Shh expression.

### proNodal, but not mature Nodal, maintains Shh

Mature Nodal is derived from its precursor protein by endoproteolytic cleavage: both forms are detected in Nodal-CM (Fig. S1). Previous studies have suggested that a subset of Nodal activities at physiological concentrations are mediated by the uncleaved precursor, proNodal (Beck et al., 2002; Ben-Haim et al., 2006; Eimon and Harland, 2002). We therefore investigated whether both the precursor and the mature protein can sustain Shh and *Gsc* expression, comparing the activity of individual forms to that of Nodal-CM.

**Fig. 3.**
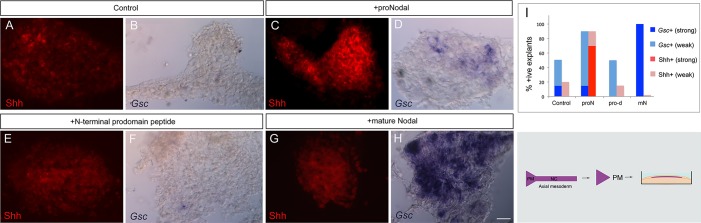
**ProNodal, but not mature Nodal, maintains Shh.** Analysis of Shh (A,C,E,G) or *Gsc* (B,D,F,H) expression in prechordal mesoderm microcultures (shown in schematic) cultured for 40 h (st13 equivalent) in control medium (A,B) or with Nodal variants (C-H). Representative examples are shown for strong (H), weak (D) or no (B,F) *Gsc* expression and for strong (C), weak (A) or no (E,G) Shh expression. (I) Quantitative analyses, showing summed data from *n*=16 explants/condition. Scale bar: 25 µm.

We first examined whether a recombinant Nodal cleavage mutant which is resistant to proprotein convertases (recombinant proNodal) can sustain Shh and *Gsc* expression in prechordal mesoderm. HH st6-7 prechordal mesendoderm explants were cultured alone, or with recombinant proNodal, until a st13 equivalent. In cultures exposed to recombinant proNodal, Shh expression is rescued in comparison to control explants, with a robustness similar to that observed for Nodal-CM (Fig. 3A,C,I, compare to Fig. 2O). *Gsc* is likewise maintained in higher numbers of explants than in controls, but not at the levels detected with Nodal-CM (Fig. 3B,D,I). By contrast, the Nodal N-terminal prodomain peptide is unable to rescue expression of Shh or *Gsc* in the prechordal mesoderm (Fig. 3E,F,I). Intriguingly, recombinant mature Nodal is likewise unable to rescue Shh expression (Fig. 3G,I) although tested over several concentrations (Materials and Methods). Conversely, mature Nodal robustly maintains *Gsc* expression, to a similar extent to that observed for Nodal-CM (Fig. 3H,I, compare to Fig. 2L). This suggests that Shh and *Gsc* are governed by distinct actions of Nodal and suggests that the duration of Shh expression in the early prechordal mesoderm might be regulated by proNodal.

### FGF signalling maintains Shh expression

We hypothesised that proNodal might exert its non-canonical effect via an interaction(s) with a second signalling pathway. Previous studies in other tissues have suggested co-operative actions between Nodal and FGF (Beck et al., 2002; Guzman-Ayala et al., 2004; Lee et al., 2011; Mathieu et al., 2004; Vallier et al., 2005; Yokota et al., 2003), and FGF receptors and signal pathway components are expressed in and around prechordal mesoderm over HH st4-st13 (Karabagli et al., 2002; Lunn et al., 2007; Nishita et al., 2011; Walshe and Mason, 2000). This led us to test the hypothesis that proNodal exerts its actions via an FGF signal pathway component.

**Fig. 4.**
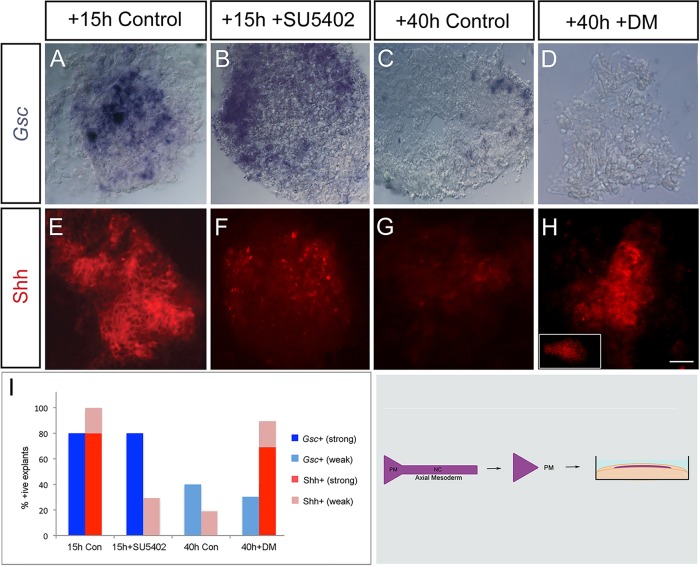
**FGF and BMP signalling pathways act antagonistically to govern Shh expression.** (A,B,E,F) Analysis of *Gsc* or Shh expression in prechordal mesoderm microcultures (shown in schematic) cultured for 15 h (st8 equivalent) in medium containing DMSO (control) or SU5402. Inhibiting FGF signalling results in no change in *Gsc* expression but prematurely downregulates Shh. (C,D,G,H) prechordal mesoderm explants cultured for 40 h (st13 equivalent) in medium containing DMSO (control) or Dorsomorphin (DM). Inhibiting BMP signalling does not affect *Gsc* expression but sustains Shh in the prechordal mesoderm. (I) Quantitative analyses, showing summed data from *n*=8-10 explants/condition. Scale bar: 25 µm.

To do so, we first asked whether general inhibition of FGF receptors results in premature Shh downregulation. HH st6 prechordal mesendoderm was cultured short term (st6 to st8) and exposed to SU5402. Inhibition of FGF receptors does not affect *Gsc* expression (Fig. 4A,B,I). By contrast SU5402 prevents Shh maintenance (Fig. 4E,F,I). To examine the involvement of FGF receptor(s) *in vivo*, we implanted beads, soaked in PBS (control) or in SU5402, adjacent to the emerging prechordal mesoderm of st4 embryos (Fig. S2A). In operated embryos exposed to control beads, and developed to st7, Shh is detected in prechordal mesoderm (Fig. S2B). Conversely, in embryos exposed to SU5402, Shh is markedly downregulated (Fig. S2D). These experiments demonstrate, again, that *Gsc* and Shh are regulated by different mechanisms in the prechordal mesoderm, and reveal that FGF receptors, like proNodal, are required to maintain Shh expression.

### ProNodal binds and activates FGFR3 to govern *Shh*

**Fig. 5.**
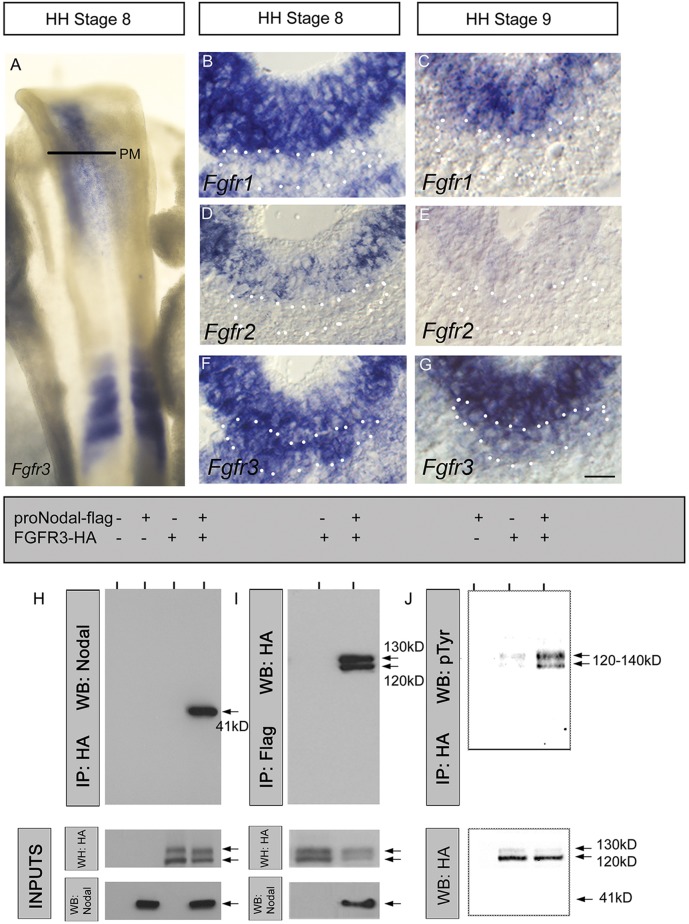
**ProNodal binds and activates FGFR3.** (A) Wholemount dorsal view of a HH st8 chick embryo after *in situ* hybridisation to detect *Fgfr3.* Expression is detected in prechordal mesoderm (PM)*.* (B-G) Transverse sections at level indicated in A, after *in situ* hybridisation to detect *Fgfr1-3*. Dotted outline demarcates prechordal mesoderm. Neighbouring sections at each stage are shown. Scale bar: 25 µm. (H) Recombinant proNodal co-immunoprecipitation with FGFR3. Expression constructs were co-transfected into HEK293T cells. Lysates were immunoprecipitated with anti-HA and analysed by western blotting (top panel) together with total lysates (‘inputs’, bottom panels: size markers as in J). Proteins were detected with indicated antibodies. A proNodal band of ∼41 kDa was clearly detected following FGFR3 immunoprecipitation. Arrows show approximate protein sizes detected. (I) FGFR3 co-immunoprecipitation with recombinant proNodal. Procedure similar to that in H but lysates immunoprecipitated with anti-FLAG. FGFR3 bands of ∼120 and 130 kDa were detected, these were also the predominant FGFR3 forms seen in the whole lysates (bottom panel; size markers as in J). (J) Analysis of FGFR3 tyrosine phosphorylation in the presence of secreted recombinant proNodal. HEK293T cells separately transfected with the indicated expression constructs were mixed and lysates immunoprecipitated as in panel H. The resulting FGFR3-containing complexes were analysed by western blotting with anti-phosphotyrosine. Phosphorylated FGFR3 protein bands were observed in the range of 120-140 kDa (top panel). A clear increase in phosphorylated FGFR3 is detected in the presence of recombinant proNodal. Total amount of FGFR3 protein in inputs verified as shown (bottom panel).

To investigate whether proNodal acts directly via FGF receptor-mediated signalling we first analysed the expression patterns of FGF receptors 1-3 (*Fgfr1-3*). *Fgfr3* is expressed in the prechordal mesoderm throughout the period that Nodal is expressed (Fig. 5A,F,G). *Fgfr1* is only weakly and transiently detected, and *Fgfr2* is not detected (Fig. 5B-E). We then determined if proNodal can bind FGF receptors, carrying out co-immunoprecipitation assays in cells co-transfected with recombinant proNodal and FGFRs or mouse Nodal and FGFRs. This revealed that proNodal can be immunoprecipitated with FGFR3 (Fig. 5H) and vice-versa (Fig. 5I). ProNodal-FGFR3 interactions can be detected both in the presence and absence of co-transfected Nodal co-receptor cripto; however, we could not detect mature Nodal-FGFR3 (data not shown) nor interactions of proNodal with either FGFR1 or FGFR2 (Fig. S3). Together, these studies suggest that proNodal binds FGFR3.

Activation of FGF receptors by ligand binding (together with heparan sulphate) results in receptor-dimerisation and leads to a conformational change and autophosphorylation of tryrosine residues in the intracellular domain (Plotnikov et al., 1999). To determine whether proNodal binding results in FGFR3 activation, we analysed the tyrosine phosphorylation status of FGFR3 in the presence or absence of recombinant proNodal (Fig. 5J). Secreted recombinant proNodal provoked a clear increase in tyrosine phosphorylation of FGFR3 (Fig. 5J) suggesting that proNodal binding can activate, or enhance activation of, the receptor. These observations strongly suggest that proNodal can bind to FGFR3 and stimulate FGFR3-mediated signalling, and raise the possibility that proNodal sustains Shh in prechordal mesoderm through this route.

**Fig. 6.**
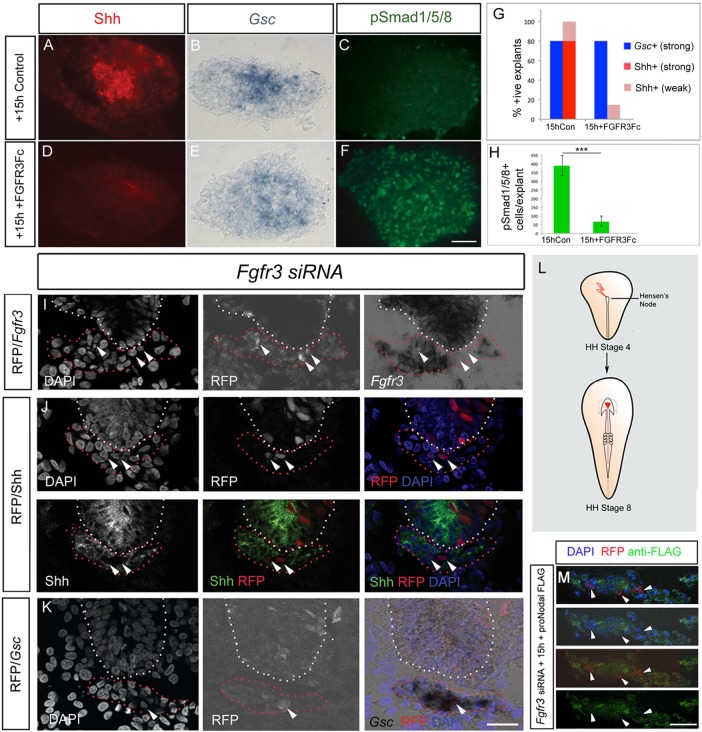
**FGFR3 signalling is required to sustain Shh in the prechordal mesoderm.** (A-F) Analysis of Shh, *Gsc* and pSmad1/5/8 expression in prechordal mesoderm explants cultured for 15 h (st8 equivalent) in control medium (A-C) or with FGFR3-Fc (D-F). FGFR3-Fc prematurely downregulates Shh, has no effect on *Gsc* expression and upregulates pSmad 1/5/8. (G) Quantitative analyses for Shh and *Gsc*, showing summed data from *n*=10 explants/condition. (H) Quantitative analyses for pSmad1/5/8, showing summed data from *n*=5 explants. ***P<0.001, unpaired Student's *t*-test, error bars show s.e.m. (I-K) Analysis of *Fgfr3* (I), Shh (J) and *Gsc* (K) after targeted electroporation of prechordal mesoderm (red dotted outline). Electroporated RFP^+^ cells downregulate *Fgfr3* (80% RFP^+^ cells are *Fgfr3*^−^, *n*=25 cells), downregulate Shh (84% RFP^+^ cells are Shh^−^, *n*=50 cells) and continue to express *Gsc* (73% RFP^+^ cells are *Gsc*^+^, *n*=30 cells). Non-electroporated cells continue to express Shh (86% RFP^−^ cells are Shh^+^, *n*=370 cells). White dotted outline indicates overlying neural tube and lumen, arrowheads indicate RFP^+^ cells. (L) Schematic, showing electroporation regime. (M) Prechordal mesoderm cells targeted with *Fgfr3* siRNA (RFP^+^ arrowheads) do not bind to proNodal FLAG (10% DAPI^+^ nuclei closely associated with anti-FLAG, *n*=30 cells). Non-targeted cells bind to proNodal FLAG (79% DAPI^+^ nuclei closely associated with anti-FLAG; *n*=277 cells). Scale bar: 25 µm.

To test this hypothesis directly, we asked whether FGFR3 is required for the action of proNodal in sustaining Shh expression in the prechordal mesoderm. In the first instance, we used FGFR3-Fc recombinant protein to out-compete FGFR3 ligands in cultured prechordal mesoderm explants. As predicted if proNodal acts via FGFR3, we detect a premature downregulation of Shh, but no alteration of *Gsc* in the presence of FGFR3-Fc (Fig. 6A,B,D,E,G). Likewise, FGFR3-Fc does not alter the ability of mature Nodal to maintain *Gsc* (not shown). To extend these findings in an *in vivo* assay, we performed targeted electroporation of *Fgfr3* siRNA/RFP to nascent (st3+ to st4) prechordal mesoderm (schematised in Fig. 6L). In such experiments, mosaic targeting of prechordal mesoderm cells was achieved. Examination of serial adjacent sections demonstrated that targeted electroporation of *Fgfr3* siRNA/RFP results in the downregulation of *Fgfr3* expression in electroporated cells at st8 (Fig. 6I). As predicted if proNodal acts via FGFR3, a premature downregulation of Shh is observed in electroporated cells (Fig. 6J), whereas non-electroporated prechordal mesoderm cells continue to express Shh as normal. *Gsc* expression is unaffected in electroporated cells, confirming that this characteristic aspect of prechordal mesoderm is unaffected by reduction in FGF signalling (Fig. 6K). Shh expression is not downregulated in similar embryos targeted with a control luciferase siRNA construct (Fig. S4). Finally, to seek evidence for a direct binding of proNodal and FGFR3 in prechordal mesoderm, isolated st6 prechordal mesoderm cells were similarly targeted with *Fgfr3* siRNA/RFP, cultured until st8 and then incubated with proNodal-FLAG. Binding was detected in non-targeted cells but not in electroporated cells (Fig. 6M). Together, these analyses indicate a requirement for FGFR3 in the action of proNodal in governing the temporal duration of Shh expression in prechordal mesoderm.

### proNodal-FGFR3 antagonises BMP signalling in prechordal mesoderm

Our studies do not distinguish whether proNodal-FGFR3 directly maintains Shh expression, or whether proNodal-FGFR3 operates indirectly, for instance, antagonising a pathway that represses Shh. Previous studies have revealed antagonistic interactions between Nodal and BMP signalling pathways (Lee et al., 2011; Lenhart et al., 2013; Yang et al., 2010; Yeo and Whitman, 2001) and BMP ligands are expressed in the prechordal mesoderm (Vesque et al., 2000). *Bmp7* is expressed particularly strongly from HH st6/7 and maintained through st8-st13 (Fig. 1F,K). However, pSmad1/5/8, a key marker of BMP signalling, is barely detectable in prechordal mesoderm prior to st8 (Fig. 1G) but thereafter robustly expressed (Fig. 1L). To begin to determine whether there might be an involvement of canonical BMP signalling in Shh regulation, we exposed prechordal mesoderm *ex vivo* to Dorsomorphin, a small molecule inhibitor that inhibits BMP type I receptors ALK2, ALK3 and ALK6 and thus blocks BMP-mediated Smad1/5/8 phosphorylation (Yu et al., 2008). Culture of prechordal mesoderm explants with Dorsomorphin does not affect the weak *Gsc* expression that is detected in such explants (Fig. 4C,D,I), in keeping with reports that suggest that its regulation does not involve BMP signalling (Vesque et al., 2000). By contrast, blockade of BMP signalling prevents normal downregulation of Shh; instead, Shh is sustained in explants cultured until st13 equivalent (Fig. 4G,H,I). The BMP inhibitor, chordin, can effect a similar rescue (Fig. 4H, inset). These data suggest that BMP signalling might downregulate Shh in prechordal mesoderm.

Our experiments show that proNodal-FGFR3 and BMP signalling exert opposite effects on Shh expression within the prechordal mesoderm, suggesting that proNodal-FGFR3 might antagonise BMP signalling, either directly or indirectly. In this scenario, elimination of proNodal-FGFR3 signalling might upregulate phosphorylated (p)Smad1/5/8. To test this prediction, we examined expression of pSmad1/5/8 in conditions where proNodal-FGFR3 signalling is prematurely eliminated. Prechordal mesendodermal explants exposed to FGFR3-Fc show robust expression of pSmad1/5/8, whereas controls do not (Fig. 6C,F,H). Similarly, *in vivo*, abolition of FGF signalling results in the upregulation of pSmad1/5/8 (Fig. S2C,E).

**Fig. 7.**
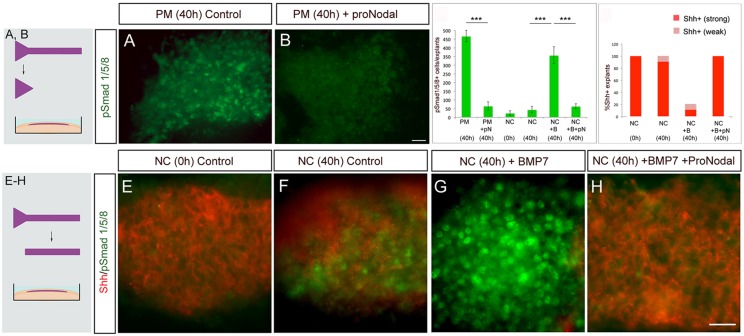
**proNodal-FGFR3 antagonises BMP signalling in prechordal mesoderm.** (A,B) Analysis of pSmad1/5/8 in prechordal mesoderm microcultures (schematic A, B) cultured for 40 h (st13 equivalent) in control medium (A) or with proNodal (B). proNodal downregulates pSmad1/5/8 expression compared with controls. (C,D) Quantitative analyses for pSmad1/5/8 (C) and Shh (D), showing summed data from *n*=5 explants/condition. ***P<0.001, unpaired Student's *t*-test, error bars show s.e.m. pN, proNodal; B, BMP7. (E-H) Double label immunohistochemical analyses of Shh (red) and pSmad1/5/8 (green) in st6 notochord microcultures (schematic E-H) after acute dissection (E) or culture to st13 (F-H) in control medium (F), with BMP7 (G) or BMP7 and proNodal (H). Shh and pSmad1/5/8 appear largely mutually exclusive. Scale bar: 25 µm.

To address whether, conversely, a sustained action of proNodal-FGFR3 signalling besides prolonging Shh is accompanied by changes to pSmad1/5/8, we examined expression of pSmad1/5/8 in cultured prechordal mesendoderm explants. Control prechordal mesendoderm was explanted at st6/7 and cultured until the equivalent of st13 (Fig. 7, top schematic). In this situation, prechordal mesendoderm explants show robust expression of pSmad1/5/8 (Fig. 7A,C). However, the upregulation of pSmad1/5/8 is significantly reduced by exposure to proNodal (Fig. 7B,C). Together, these experiments show that proNodal-FGFR3 can antagonise BMP signalling in the prechordal mesoderm.

To corroborate a cross-talk between proNodal-FGFR3 and BMP signalling pathways in Shh regulation, we established an *ex vivo* assay to independently examine the effects of proNodal and BMP signalling. Notochord, a tissue that closely approximates the prechordal mesoderm, and that expresses Shh but not Nodal and BMPs, was explanted and cultured (Fig. 7, bottom schematic) alone, with BMP7, or with BMP7 and Nodal. At the onset of culture, control explants express Shh but little or no pSmad1/5/8 (Fig. 7C-E). Control explants cultured to st13 equivalent express Shh and minimal pSmad1/5/8 (Fig. 7C,D,F). Sister cultures exposed to BMP7 show a loss of Shh expression and elevated levels of pSmad1/5/8 (Fig. 7C,D,G). By contrast, when added together with proNodal, BMP7 fails to upregulate pSmad1/5/8 and Shh expression remains high (Fig. 7C,D,H). Western blot analysis confirms that BMP7-conditioned medium activates pSmad1/5/8 in the absence, but not in the presence, of exogeneous Nodal (Fig. S5). These results show that Nodal blocks BMP-mediated Smad1/5/8 phosphorylation independently of intracellular physical interactions and/or heterodimer formation.

Together, these experiments suggest that proNodal-FGFR3 governs Shh duration by repressing canonical BMP signalling to prevent premature BMP signalling in the prechordal mesoderm. This is consistent with a model in which local BMPs are poised to rapidly silence Shh once endogenous Nodal-FGFR3 signalling is downregulated.

## DISCUSSION

In many regions of the embryo the response of cells to Shh is at least in part a function of the duration of their exposure to the signal (Dessaud et al., 2007; Harfe et al., 2004). In the prechordal mesoderm, the window of Shh expression is crucial to the development of overlying forebrain cells (Cordero et al., 2004; Mercier et al., 2013; Ohyama et al., 2005; Zhang et al., 2006). Shh is required through stages at which the prechordal mesoderm induces RDVM cells, and initiates patterning of the telencephalon, pre-optic regions and anterior/tuberal hypothalamus (Dale et al., 1999; Pearson and Placzek, 2013; Seifert et al., 1993) before being downregulated. Here, we describe signalling interactions that govern the timing of Shh expression in the chick prechordal mesoderm. We demonstrate that Nodal governs the duration of Shh expression by antagonising BMP7 and pSmad1/5/8. In contrast to *Gsc* regulation, the maintenance of Shh in prechordal mesoderm appears to be mediated by the proNodal precursor, which acts via FGFR3 activation and an as-yet-unknown non-canonical pathway. Together these findings suggest a novel signalling route for proNodal, via FGFR3.

### Temporal regulation of BMP signalling governs Shh duration

A number of lines of evidence suggest that maintenance of Shh depends on an active Nodal signalling system that operates by antagonising BMP signalling. Exposure of prechordal mesoderm explants to the Nodal antagonist CerS or to an anti-Nodal antibody results in premature downregulation of Shh. Conversely, exposure of prechordal mesoderm explants to recombinant proNodal beyond st11-12 results in prolonged expression of Shh and a failure to upregulate pSmad1/5/8. The simplest explanation of this result is that Nodal normally prevents activation of the BMP signalling pathway. In support of this, the downregulation of *Nodal in vivo* is followed immediately by the phosphorylation of Smad1/5/8 and the downregulation of Shh. Finally, whereas BMP7 extinguishes Shh expression in notochord explants, exposure to recombinant proNodal and BMP7 rescues Shh expression. Thus, proNodal is both necessary and sufficient to counteract the BMP-mediated downregulation of Shh.

What is the significance of Shh downregulation? By the time that Shh is downregulated, prechordal mesoderm is in register with the posterior (mamillary) hypothalamus (Dale et al., 1999; Pearson and Placzek, 2013; Seifert et al., 1993). At this time, Tbx2 and Tbx3 are mediating downregulation of Shh in the hypothalamic floor (Manning et al., 2006; Trowe et al., 2013), an event that is crucial for the development of Emx2^+^ mamillary progenitor cells. Forced prolonged exposure of hypothalamic floor plate cells to Shh prevents their normal progression to Emx2^+^ mammillary progenitors (Manning et al., 2006). We therefore predict that the downregulation of Shh in prechordal mesoderm is important for the development of the hypothalamic mamillary region. A second possibility is that Shh downregulation supports the subsequent fate of the prechordal mesoderm itself. From HH st8, cells at the lateral edges of prechordal mesoderm cells begin to disperse, giving rise to ocular muscles and thymic myoid cells (Couly et al., 1992; Jacob et al., 1984; Seifert and Christ, 1990). We first detect pSmad1/5/8 at the lateral edges of the prechordal mesoderm at HH st8, and from this stage, the prechordal mesoderm begins to narrow. Potentially, then, a downregulation of Shh, from lateral to medial, occurs over HH st8-st12, enabling such migration and/or differentiation programmes. Finally, Shh downregulation might eliminate some prechordal mesoderm cells: previous studies have suggested that Shh plays an autocrine role, supporting prechordal mesoderm cell survival (Aoto et al., 2009).

### ProNodal acts via FGFR3 to maintain Shh

In contrast to its ability to maintain *Gsc* expression in explant cultures, mature Nodal does not appear to maintain Shh expression. Conversely, a Nodal cleavage mutant is able to robustly maintain Shh expression. Thus, although we cannot exclude that a relative instability of mature Nodal (Le Good et al., 2005) masks its ability to sustain Shh, our studies suggest that the maintenance of Shh in prechordal mesoderm is mediated by the proNodal precursor. Our studies add to a growing body of evidence that proNodal-like ligands exhibit activities that cannot easily be mimicked by the N-terminal prodomain or the mature ligand (Tian et al., 2008). How might proNodal antagonise BMP signalling? Previous studies have shown that Nodal- and BMP-activated Smads can compete for their common partner (Candia et al., 1997). However, in prechordal mesoderm, we detect little or no pSmad1/5/8 in the presence of proNodal, strongly suggesting that BMP7 signalling is inhibited upstream of Smad4. ProNodal is also unlikely to antagonise BMP through the formation of Nodal-BMP heterodimers: recombinant proNodal can counteract the effect of BMP7 and block pSmad1/5/8 in prechordal mesoderm, even if the two ligands are added independently. Biochemical evidence suggests that under these conditions, no BMP7-Nodal interactions occur, even though BMP7 and Nodal can heterodimerise when co-expressed in the same cells (Yeo and Whitman, 2001; data not shown).

Instead, our experiments show that the proNodal-mediated maintenance of Shh is not inhibited by the ALK inhibitor SB431542, suggesting that in this context, proNodal activates a non-canonical pathway. Previous cell culture studies have shown that proNodal is more stable than mature Nodal (Le Good et al., 2005), correlating with reduced endocytosis and increased accumulation at the cell surface (Blanchet et al., 2008). Since Smad2/3, the mediator of canonical Nodal-ALK signalling, is activated by endosomal signalling platforms (Constam, 2009), one might expect that proNodal can preferentially activate alternative pathways. Our analyses suggest that proNodal acts via FGFR3. First, *Fgfr3* is expressed in prechordal mesoderm and knockdown of *Fgfr3* eliminates the ability of proNodal to bind to prechordal mesoderm. Second, a proNodal-FGFR3 interaction is detected after co-transfecting FGFR3 and wild-type Nodal (which produces both proNodal and mature Nodal in the supernatant). ProNodal promotes FGFR3 under these conditions but, as yet, the pathway triggered by a proNodal-FGFR3 interaction remains unclear: MAPK is not phosphorylated, and indeed, we find no evidence for pMAPK in prechordal mesoderm *in vivo* at the relevant stages (not shown). Overall, these results suggest that the activity of proNodal in prechordal mesoderm is mediated by an unknown signalling pathway, triggered upon activation of FGFR3. Previous reports in *Xenopus*, using loss-of-function analysis, have indicated that *Xenopus* Nodal related 3 (Xnr3) and FGFR1 might act synergistically (Yokota et al., 2003). Our experiments add significantly to this, and indicate a direct interaction between Nodal and FGF signalling pathways, although the direct interaction we see occurs between proNodal and FGFR3, not FGFR1.

Our investigations suggest that the interaction of proNodal and FGFR3 is functionally relevant. *In vitro*, blockade of FGFR signalling resulted in a premature increase in pSmad1/5/8, and premature downregulation of Shh within the prechordal mesoderm. Similarly, *in vivo* Shh is extinguished in cells electroporated with *Fgfr3* siRNA. Although there are no current reports that the forebrain is abnormally specified in *Fgfr3*^−/−^ mice, to date such studies have focused on wider phenotypic analyses (Colvin et al., 1996; Deng et al., 1996). However, both Nodal and FGF signalling components have been implicated in holoprosencephalic phenotypes (Mercier et al., 2013; Monuki, 2007; Taniguchi et al., 2012): our experiments support the idea that they might mediate their effects in part through misregulation of prechordal mesodermal expression of Shh.

### Separate and sequential modes of Nodal function in prechordal mesoderm

Nodal plays an essential role in embryonic mesoderm and axis formation (Conlon et al., 1994; Zhou et al., 1993). Our results indicate that this role does not operate through one simple mechanism. Many studies have indicated that Nodal acts through the canonical ALK-Cripto-Smad2,3 pathway to govern early axial mesoderm cell specification. In zebrafish, the Nodal-Smad2/3 pathway establishes notochord or prechordal mesoderm fate, depending on distinct signalling thresholds (Gritsman et al., 2000; Lowe et al., 2001; Schier and Shen, 2000). In mouse, hypomorphic mutations in the *Nodal* gene (Dougan et al., 2003; Norris et al., 2002), loss of Cripto (also known as Tdgf1) (Chu et al., 2005; Ding et al., 1998), reductions in the gene dosage of Smad2 and -3 (Vincent et al., 2003), ablation of the transcriptional Smad2/3 coactivator Foxh1 (Hoodless et al., 2001; Yamamoto et al., 2001) and loss of PACE4 (also known as Pcsk6), a Nodal convertase (Constam and Robertson, 2000), all inhibit or impair the induction of prechordal mesoderm progenitors. Our studies, similarly, show that Nodal acts via an ALK-dependent route to govern *Gsc* expression, and suggest that this is mediated by mature Nodal. By contrast, our studies illustrate that an ALK-independent route of Nodal signalling is essential for later prechordal mesoderm specification, in particular Shh maintenance, and that this is mediated by proNodal-FGFR3.

Our studies suggest, similarly, that the ALK-dependent and ALK-independent functions of Nodal can exert very different effects on BMP signals. For instance, in early gastrulation, Nodal induces BMP signals, and the synergism of the two signals is crucial for the general induction and patterning of mesoderm (Ben-Haim et al., 2006; Brennan et al., 2001; Guzman-Ayala et al., 2004). In this context, proNodal signalling appears to play a pivotal role, but acts via Cripto-independent ALK signalling (Ben-Haim et al., 2006). Our studies reveal that proNodal plays an additional role within later prechordal mesoderm specification, but in this context, it antagonises BMP activity in an ALK-independent manner. This data adds to the growing body of evidence that Nodal can both stimulate or inhibit BMP signalling, depending on the developmental context.

In conclusion, our finding that proNodal operates via FGFR3 activation to sustain Shh suggests an additional level of complexity in Nodal signalling and in prechordal mesoderm specification. Several reports suggest a synergism between Nodal and FGF activities that relies on ALK activation (Beck et al., 2002; Ben-Haim et al., 2006; Guzman-Ayala et al., 2004; Vallier et al., 2005). Our studies show that Nodal and FGF activities can synergise in an ALK-independent manner to effect an inhibitory crosstalk between the Nodal and BMP pathways in the prechordal mesoderm and govern Shh signalling. Given that Nodal and FGFR3 overlap elsewhere in the body, our observations raise the intriguing possibility that the novel antagonism we describe between proNodal-FGFR3 signalling and canonical BMP signalling might be more broadly relevant.

## MATERIALS AND METHODS

### Chick strains

Bovan brown chick embryos were obtained from Henry Stewart (MedEggs, Heath Farm House, Norfolk, UK). All experiments involving live chick embryos conformed to the relevant regulatory standards.

### Tissue dissection and explant culture

Prechordal mesoderm and notochord were identified and dissected as described (Vesque et al., 2000). Explants were cultured for set periods in explant medium, fixed in cold 4% paraformaldehyde and examined by *in situ* hybridisation or immunohistochemistry. SU5402 (Calbiochem) was used at 20 µM; SB431542 (Sigma) at 25 µM; Human FGFR3-Fc(IIIc) (R&D Systems) at 600 ng/ml; Dorsomorphin (Sigma) at 1 µM; CerS-CM (see below) at 1×; anti-mouse Nodal (1:500; Yan et al., 2002).

### Immunocytochemistry

Fixed tissues were sectioned and incubated with primary antibodies: anti-Shh 5E1 (1:50; Developmental Studies Hybridoma Bank); anti-pSmad 1/5/8 (1:100-1:500; Cell Signaling Technologies, cat. no. 9511). Alexa 488 and 594 conjugated secondary antibodies were used (1:500; ThermoFisher Scientific/Molecular Probes, cat. nos. A11001, A11034 and A11005). Slides were mounted in Vectashield (Vector Laboratories) and analysed.

### *In situ* hybridisation

*In situ* hybridisation was carried out on embryos and explants using standard techniques (Vesque et al., 2000).

### Cell transfection and supernatant production

Supernatants were collected in serum-free Opti-MEM (Gibco) 3-4 days after lipofectamine-transfection of constructs: pCS2+ Flag-Cerberus short (Piccolo et al., 1999); pdMycBMP7 (Basler et al., 1993); pcDNA3-mouseNodal, pCS2+FlagProNodal; pCS2+FlagNprodomain into HEK293T cells. pCS2+FlagNprodomain was generated by subcloning the *Eco*RI-*Xho*I insert of MT21-FlagNpro (Le Good et al., 2005). Secreted proteins were confirmed by SDS-PAGE and western blotting. Empty vectors or irrelevant gene constructs were transfected for controls, and used to standardise DNA transfected.

For co-immunoprecipitation experiments, HEK293T cells were co-transfected with a total amount of DNA not exceeding 10 μg/10 cm dish. For cell mixing experiments, cells were transfected with single plasmids in individual dishes, dissociated 5 h later in PBS-Ca/Mg, pelleted, mixed with alternate transfected cells and replated. Mixed cells were cultured for 40 h, lysed and proteins analysed by immunoprecipitation/western blotting. FGFR1-GFP, FGFR2 and FGFR3-HA plasmids were kindly provided by P. Maher, J. Heath and E. Liboi, respectively (Dunham-Ems et al., 2006; Lievens and Liboi, 2003).

Protein supernatants produced in transient transfections were concentrated 10-fold on Centricon-plus20 10,000 MW cut-off columns (Millipore) and diluted in explant medium to 1× unless stated. Nodal-CM supernatant was used at 3× to antagonise 1× BMP7 supernatant or endogenous BMP7 in explant assays. Individual Nodal forms were used at concentrations standardised to Nodal-CM forms by western blot comparison. Mature Nodal was therefore used at 100-150 ng/ml (and exceptionally at 2.5 μg/ml in excess condition) and was normalised accordingly. FLAG-tagged Nodal prodomain was added at ∼50 ng/ml.

### Co-immunoprecipitation and western blot analysis

Reversible cell surface crosslinking was carried out 24-48 h after transfection using 0.5 mM DTSSP. Cells were lysed in 50 mM Tris-Cl pH 7.5, 150 mM NaCl, 1 mM EDTA, 1% Triton X-100 and protease inhibitor cocktail (Complete Mini, Roche), 1 mg total protein was used. Co-immunoprecipitation (CoIP) assays were performed for 3 h at 4°C using 50 µl protein G Dynabeads (Invitrogen) pre-bound to either 1 μg rat anti-HA 3F10 MAb (Roche Diagnostics, cat. no. 1867423001) or 2 μl rabbit anti-Nodal antiserum (Yan et al., 2002) or FLAG M2-conjugated beads (Sigma Aldrich). Complexes were eluted from Dynabeads by heating with 1× LDS loading buffer (Invitrogen). Beads bound to FLAG were eluted by FLAG peptide competition. Crosslinking was reversed by boiling in 1× reducing agent (Invitrogen) and 2.5% β-mercaptoethanol. For analysis of FGFR3 phosphorylation status, heparin (10 u/ml, Sigma) was added to the cells for the last 16 h of culture. Sodium pyrophosphate (30 mM, Sigma) and sodium orthovanadate (1 mM, Sigma) were added to lysis buffer and washes done in Tris-buffered saline. Following IP with anti-HA, phosphorylated FGFR3 was detected with anti-phosphotyrosine 4G10 (1:2000; Fisher Scientific, cat. no. MZ05321X), *n*=4-5 for each coIP/phosphorylation assay. Proteins were run on SDS-PAGE gels (NuPAGE, 4-12% Bis-Tris; Invitrogen) under reducing conditions. The following antibodies were used for western blot analysis: anti-FLAG (1:1000; Sigma Aldrich, cat. no. F3165-0.2mg), anti-mouse Nodal (1:4000; Yan et al., 2002); anti-myc (A14) (1:400; Santa Cruz Biotechnology, cat. no. Sc-122), anti-pSmad1/5/8 (1:1000; Cell Signaling Technology, cat. no. 9511), anti-HA (1:1000; Roche Diagnostics; cat. no. 1867423001), anti-phosphotyrosine 4G10 (1:2000; Fisher Scientific, cat. no. MZ05321X), anti-GFP (1:1000; Becton Dickinson, cat. no. 632460), anti FGFR2 C-17 (1:200; Santa Cruz Biotechnology; cat. no. Sc-122) and anti-actin AC-40 (1:1000; Sigma Aldrich, cat. no. A3853). Peroxidase-conjugated anti-IgG secondary antibodies were used at 1:5000 (Jackson ImmunoResearch Laboratories, PA, USA, cat. nos. 115-035-146 and 111-035-144) and detected with ECL (Amersham Pharmacia Biotech, UK) or at 1:20,000 with Supersignal West Dura (Pierce).

### *In ovo* electroporation

Chick siRNA constructs for *Fgfr3* and luciferase (control) were generated using methods described (Das et al., 2006). PM was targeted by introducing the constructs to Hensen's node at st3+/4 using standard *in ovo* electroporation techniques (Gray and Dale, 2010). Electroporated chick embryos were allowed to develop overnight then fixed and analysed.
